# Exploring Lifestyle Factors and Treatment Adherence among Older Adults with Hypertension Attending a Mobile Health Unit (MHU) in a Rural Area of Central Portugal

**DOI:** 10.3390/nu16081112

**Published:** 2024-04-10

**Authors:** Cátia Pinto, Cláudia Chaves, João Duarte, António Raposo, Renata Puppin Zandonadi, Sara Monteiro, Edite Teixeira-Lemos

**Affiliations:** 1 Câmara Municipal de Castro Daire, Rua Dr. Pio Figueiredo, No. 42, 3600-126 Castro Daire, Portugal; catiapinto-enf@hotmail.com; 2 ESSV, Centre for Studies in Education and Innovation (CI&DEI), Polytechnic University of Viseu, 3504-510 Viseu, Portugal; cchaves@essv.ipv.pt (C.C.); duarte.johnny@gmail.com (J.D.); 3 CBIOS (Research Center for Biosciences and Health Technologies), Universidade Lusófona de Humanidades e Tecnologias, Campo Grande 376, 1749-024 Lisboa, Portugal; 4 Nutrition Department, Faculty of Health Sciences, University of Brasília, Campus Universitário Darcy Ribeiro, Brasilia 70910-900, Brazil; renatapz@unb.br; 5 Center for Health Technology and Services Research of the Health Research Network (CINTESIS@RISE), Department of Education and Psychology, University of Aveiro, 3810-193 Aveiro, Portugal; smonteiro@ua.pt; 6 CERNAS Research Centre, Polytechnic University of Viseu, 3504-510 Viseu, Portugal

**Keywords:** hypertension, dietary habits, diet, lifestyle, treatment

## Abstract

This cross-sectional and analytical study aimed to characterize a sample of hypertensive older adults attending a Mobile Health Unit (MHU) in a rural area of central Portugal according to their lifestyle and to analyze the impact of lifestyles on treatment adherence. The sample comprised 235 Portuguese hypertense patients, mainly females (63.8%) with a mean age of 75 years (±8.14 years) and low level of education. The data collection was carried out through a questionnaire consisting of sociodemographic questions, dietary variables, an Alcohol Dependence Questionnaire, an International Physical Activity Questionnaire (Short Version), a Nutrition Health Determination Questionnaire, a Self-Care with Hypertension Scale, and an Adherence to Treatments Measurement Scale. Only 34.5% of the hypertensive patients have controlled blood pressure values (28.2% men and 38% women). However, more than half (56.2%) of the hypertensive patients are classified as adherent to therapeutic measures. The hypertensive individuals, who present higher levels of adherence to the treatment, do not present alcohol dependence, are frequent consumers of aromatic herbs, sporadically consume salt, present good nutritional health, and practice moderate physical activity. The predictor variables for treatment adherence are the self-care dimensions general dietary (*p* = 0.001), specific dietary (*p* = 0.034), physical activity (*p* = 0.031), and antihypertensive medication intake (*p* < 0.001). Hypertensive patients with healthier lifestyles present better levels of treatment adherence. Therefore, promoting physical activity and healthy dietary practices is necessary to improve treatment adherence and increase antihypertensive treatment’s effectiveness.

## 1. Introduction

The burden of chronic diseases in society has increased. The leading causes of preventable morbimortality are related to unhealthy lifestyles and behavior, most of which can be avoided. Cardiovascular diseases are the main cause of morbimortality in contemporary society, with arterial hypertension (AHT) being one of the main risk factors [1]. AHT is defined by systolic blood pressure (SBP) ≥ 140 mmHg and/or diastolic blood pressure (DBP) ≥ 90 mmHg in subjects aged ≥18 years [1,2]. Studies have referred to age, excess weight, excessive alcohol consumption, sedentarism, dyslipidemia, diabetes mellitus, and high sodium content in food as factors associated with increased blood pressure [1]. Control of risk factors is crucial for effective control of blood pressure [3].

It is estimated that 1.3 billion adults present AHT worldwide in 2019 [2]. The World Health Organization (WHO) estimated a prevalence of AHT of 32% for Portugal, 37% for males and 28% for females [4]. However, the prevalence seems to be higher in Portugal than the WHO’s estimates. A study in Portugal with 5023 individuals aged between 18 and 90 years showed that the prevalence of AHT was 42.1%, with the highest percentage among males (49.5% vs. 38.9%) [5]. Similar results were found in another study in Portugal with adults who attended primary healthcare services (42.62% of adults with AHT—43.09% in males vs. 42.19% in females) [6]. The Portuguese Hypertension and Salt Study conducted in 2011 and 2012 found a prevalence of 42.2% of AHT, which was also higher in males (44.4% vs. 40.2%) [7].

As a chronic disease, AHT has no cure, but in most cases, it is controllable. Adopting a healthy lifestyle can prevent the onset of the disease, and an early diagnosis and follow-up may reduce the risk of cardiovascular diseases [2,8,9]. Thus, arterial hypertension treatment is based on pharmacological treatment and embracing healthy lifestyles such as dietary care and physical exercise. Controlling hypertension in different populations has been linked to several factors, including patient awareness of hypertension, attitudes toward medication, and adherence to antihypertensive treatment [1,10,11]. The term ‘adherence’ refers to how well people with hypertension follow the recommended therapeutic and behavior changes to control their blood pressure. Thus, health services have a difficult task in promoting adherence among individuals with hypertension [3].

The increasing prevalence of chronic conditions in Portugal has profound consequences on the national healthcare service, requiring a shift in the current healthcare model. The primary approaches to managing these conditions involve population-wide screening for hypertension, monitoring patients over time, and establishing primary healthcare systems within the community. Rural places suffer from depopulation, and dispersed settlements and limited access generally result in poorer health outcomes. To address the difficulties experienced by the population, particularly older adults who are more vulnerable and face mobility challenges and isolation, some municipalities have created Mobile Health Units (MHUs). These MHUs offer screenings for diabetes and hypertension, manage therapeutic regimens, monitor individual vaccination plans, and provide education to promote behavioral improvements for better health. They represent an innovative approach to address healthcare disparities and improve health outcomes in underserved populations. While there have been numerous studies on hypertension and lifestyle factors, this study focuses on a specific population accessing healthcare through an MHU, which represents an underexplored area in the existing literature. Consequently, information is needed about the lifestyle characteristics of hypertensive patients and treatment adherence patterns of hypertensive patients who utilize the MHU. Additionally, there needs to be more understanding of the influence of lifestyles on treatment adherence among Portuguese patients with hypertension. Therefore, the following objectives were delineated to address this inquiry: (i) characterize the hypertensive population attending the MHU in a rural area of central Portugal according to their lifestyle and (ii) analyze the impact of lifestyles on treatment adherence.

## 2. Materials and Methods

A cross-sectional and analytical study was performed in a non-probability convenience sample of 235 Portuguese hypertense patients from the municipality of Castro Daire who use the Mobile Health Unit (MHU). The inclusion criteria were to reside in the municipality of Castro Daire (a rural area in the center of Portugal); have a clinical diagnosis of arterial hypertension, according to norm 020/2011, updated in 2013, of the Directorate General of Health Services (DGHS) [12]; age ≥ 18 years; enrolled in the MHU in the functional units of the Castro Daire Health Centre; and voluntarily accept to participate in the study. Exclusion criteria involved people with dementia and communication difficulties.

The data collection instrument used was a questionnaire, consisting of sociodemographic variables and dietary variables (perception of the type of diet, use of salt and herbs, and the consumption of food with a high salt content), the Determine Your Nutritional Health [13] questionnaire, the Questionnaire on Alcohol Dependence (CAGE) [14], the International Questionnaire on Physical Activity (IPAQ—Short Version) [15], the Self-Care with Hypertension Scale [11], and the Measurement of Adherence to Treatment Scale (MAT) [16]. Once the participants had given their informed consent, experienced investigators were available to assist in filling out the questionnaires. This procedure ensured that all participants could provide their responses, regardless of their literacy levels. The investigators read the questions aloud and marked the participants’ responses. This methodology aimed to maintain consistency and accuracy in data collection while catering to participants’ needs at different literacy levels.

The questionnaire on alcohol dependence—CAGE [14]—consists of four questions that aim to evaluate the main symptoms of alcohol dependence (Cut, Annoyed, Guilty and Eye-opener). Its briefness and simplicity of response characterize it. Scores of two or more positive responses suggest alcohol dependence.

Physical exercise was evaluated using the International Physical Activity Questionnaire—IPAQ (Short Version) [15]—which consists of seven questions about the activities carried out within the last seven days before administering the questionnaire. The estimate of energy expenditure follows the IPAQ Data Processing and Analysis Guidelines [17].

The Determine Your Nutritional Health, developed by the American Academy of Family Physicians, American Dietetic Association, and National Council on the Aging [13], aims to determine the state of nutritional health. It is a questionnaire consisting of ten questions. The nutritional risk is calculated through the sum of the positive responses; each positive response has a different score. Yes corresponds to two points in questions 1, 3, 4, 5, 9, and 10, to three points in question 2, to four points in question 6, and to one point in questions 7 and 8. The total scores’ sum make it possible to classify nutritional health: 0 to 2 points suggest good nutritional health; 3 to 5 points suggest moderate nutritional risk; and 6 points or more suggest high nutritional risk.

To evaluate self-care with hypertension, the Self-Care with Hypertension Scale by Ribeiro [11] was used to evaluate the adherence to the therapeutic regimen in people with A, considering the various aspects of the AHT treatment and its multidimensionality. The scale is parameterized on days per week, on a scale of 0 to 7, corresponding to the behaviors adopted in the last seven days, with zero being the least desirable situation and seven being the most favorable.

The general dietary subscale refers to eating habits not specific to any pathology. It comprises three items, and the result is obtained by the respective mean expressed in days per week. The specific dietary subscale refers to eating habits that raise blood pressure values. This consists of 8 items, where the score is reversed. In other words, the score is inverted (0 = 7; 1 = 6; 2 = 5; 3 = 4; 4 = 3; 5 = 2; 6 = 1; 7 = 0), and the result is obtained through the mean and expressed in days per week. The subscale of physical activity consists of two items, and the result is obtained by the respective mean expressed in days per week. The subscale of smoking habits consists of three items and is evaluated independently. In this study, the subscale of drugs, which appear in the Self-Care Activities with Diabetes Scale [18], was included. The latter consists of two items on antihypertensive medication intake; the result is obtained by the respective mean and is expressed in days per week. Generally, the adherence level, by size, is obtained by the sum of the items and divided by the respective number; the results (means) are expressed in days per week.

The Measurement of Adherence to Treatment Scale (MAT) was created by Morisky, Green, and Levine [19], translated, adapted, and validated for Portugal by Delgado and Lima [16]. MAT comprises seven items, answered on a Likert scale of 6 points, ranging from 1 = Always to 6 = Never. The sum of the values of each item and its division by the number of items makes it possible to obtain a level of adherence to the treatments. The score ranges from 1 to 6. It can also be converted into a dichotomous scale, with the conversion being as follows: never (6) and rarely (5) of the Likert scale change to no (1) of the dichotomous scale; sometimes (4), often (3), almost always (2) and always (1) of the Likert scale change to yes (0) of the dichotomous scale. In this case, the score ranges from 0 to 1. In both cases, higher values translate into a higher level of adherence.

The descriptive and inferential statistical treatment was processed through the SPSS program (Statistical Package for the Social Science) version 23.0 (2015) for Windows. Regarding the descriptive statistics, absolute and percentage frequencies, measures of central tendency, dispersion measures, form measures, and association measures were established. In the inferential analysis, parametric and non-parametric statistics were used, applying the Mann–Whitney U test, the Kruskal–Wallis test, the one-way analysis of variance ANOVA, the Tukey Post hoc tests, and simple linear regressions.

## 3. Results

Most of the sample was female (63.8% vs. 36.2%). The mean age was 75 ± 8.14 years and most were married or in a civil partnership (62.6%), had completed the 1st cycle of basic education (58.3%), were retired (87.7%), and resided in the village (Table 1).

In the total of hypertensive patients, only 34.5% had controlled blood pressure, meaning they had SBP values lower than 140 mmHg and/or DBP lower than 90 mmHg.

As for gender, this percentage is more noticeable in females than males (38.0% and 28.2%, respectively) (Table 2). Concerning the intensity of physical activity practiced by hypertensive people, 60.0% report practicing high-intensity physical exercise, 22.1% moderate intensity, and 17.9% practice low-intensity physical exercise, which is considered inactive or sedentary. Comparing genders, females are the most active (61.3%) and males are the most sedentary (20.0%) (Table 2).

Regarding smoking habits, it is verified that the majority of the sample has never smoked (88.5%); 9.4% smoked over two years ago; 1.3% smoked 1–2 years ago, and 0.9% currently smoke, that is, they are active consumers. Stratifying by gender, it is found that females have lower smoking habits, since 99.3% state they have never smoked and only 0.7% consumed tobacco two years ago. Currently, there are no female smokes, while 69.4% of males say they have never smoked, 24.7% state they smoked two years ago, 3.5% say that their last consumption was 1–2 years ago, and 2.4% currently smoke (Table 2).

Overall, 56.2% of hypertensive patients adhere to the therapeutic measures, while 43.8% do not adhere to the treatment. Regarding gender, we observe that the male gender adheres more to the treatment than the female gender (58.8% vs. 54.7%, respectively) (Table 2). Regarding nutritional health, 63.4% of the individuals present ‘good nutritional health’. There is a greater number of individuals within the male gender that present ‘high nutritional risk’ (16.5% vs. 9.3%) and ‘moderate nutritional risk’ (28.2% vs. 22.7%) when compared to the female gender (Table 2).

According to Table 3, the type of diet that has the highest percentage is ‘moderately healthy’ (70.2%), followed by ‘not very healthy’ (15.7%), ‘healthy as recommended’ (12.3%), and ‘very healthy’, with only 1.7%. No individual reports having an ‘unhealthy’ diet. Regarding gender, in general, females report having a healthier diet. Only in the item of very healthy diet do males have a higher percentage (2.4% vs. 1.3%). Regarding the seasoning used in the preparation of meals, 49.4% refer to never using aromatic herbs in meals, and, in contrast, 78.3% always use salt. Regarding coffee consumption, 52.8% of individuals report never drinking coffee. Almost half of the individuals (45.5%) mention ‘never’ having consumed alcoholic beverages, followed by individuals who report ‘always’ consuming alcoholic beverages (23.0%). As for sausage consumption, 48.5% say their intake is ‘rare’ (males 50.6% and females 47.3%). Concerning the consumption of smoked ham, it is found that most people ‘rarely’ eat it (45.5%). More than half of the people report not consuming lupin beans (64.7%), and 23.0% mention ‘never’ consuming olives. According to the CAGE questionnaire, 7.2% of hypertensive individuals present alcohol dependence (12.5% in males and 1.6% in females).

The Self-Care with Hypertension scale has five dimensions: general dietary, specific dietary, physical activity, medication intake, and smoking habits. In the subscale of general dietary, it is verified that the minimum value is 0.67 days and the maximum is 7 days (4.74 ± 1.39 days). Thus, on average, hypertensive people practice a nonspecific healthy diet 4.74 days/week. For the specific dietary subscale, a minimum value of 0.75 days and a maximum of 4 days (2.18 ± 0.55 days) is observed. That is, hypertensive individuals eat, on average, food that potentially increases blood pressure 2.18 days/week. As for the subscale of physical activity, it is verified that the minimum value is 0 days and the maximum is 7 days (3.34 ± 2.38 days). On average, hypertensive individuals practice exercise 3.34 days/week. With the subscale of medication intake, the minimum value is 1 day and the maximum is 7 days (6.67 ± 0.92 days). On average, people with hypertension take antihypertensive drugs 6.67 days/week (Table 4).

Analyzing whether alcohol dependence affects treatment adherence, the Mann–Whitney U test (UMW) was performed. It is verified that the mean ranks are higher (MO = 63.53) in non-alcohol-dependent individuals. However, the differences found are not statistically significant (*p* = 0.536). Concerning the consumption of aromatic herbs in treatment adherence, it is verified that the mean orders are higher (MO = 124.68) in the individuals who state that they ‘frequently/always’ consume aromatic herbs, with no statistically significant differences (*p* = 0.518). For salt consumption, the mean orders are higher (MO = 153.00) in those who say they ‘never/sporadically’ consume salt, and the differences found are not significant (*p* = 0.100). The Kruskal–Wallis test (KW) was used to determine that hypertensive individuals with ‘good’ nutritional health are the ones that show greater treatment adherence since they present the highest mean order (MO = 120.58). However, the differences between nutritional health and treatment adherence are not significant (*p* = 0.726). Concerning physical activity, hypertensive individuals with moderate physical activity are the ones that present a greater adherence to treatment, since they have the highest mean order (MO = 128.02), but without significant differences (*p* = 0.266) (Table 5).

All the dimensions of self-care have proven to be predictors of treatment adherence. Thus, the general dietary subscale accounts for 14% compliance (t = 3.365, *p* = 0.001). Regarding the specific dietary subscale, it explains 8.7% of the adherence and presents a negative correlation (t = −2.129; *p* = 0.034). In other words, the higher the intake of food that promotes AHT, the lower the treatment adherence. The practice of physical activity explains 8.9% of the adherence (t = 2.176, *p* = 0.031). Taking antihypertensive medication explains 73.3% of the treatment adherence (t = 17.978, *p* < 0.001).

## 4. Discussion

While there have been previous studies on hypertension and lifestyle factors, the present study’s emphasis on the population accessing healthcare through the MHU in a rural setting adds a unique dimension to the existing literature. The sample consisted of 235 people, mostly females (63.8%) aged 75 ± 8.14 years. Similar results were found by Ferreira et al. [20], with a sample of 332 people with arterial hypertension; the female gender was more representative (59.3%), and the age was 64.33 ± 12.38 years. The age distribution reflects the aging population in Portugal’s interior regions, highlighting the importance of addressing health concerns specific to older adults.

The Mobile Health Unit (MHU) is a strategy implemented by a collaboration between the local health units and the Municipality of Castro de Aire (center of Portugal). The UMS focuses on identifying the health needs of the individual, family, group, or community; planning care; coordinating and developing interventions; and analyzing and interpreting results to achieve health gains. In the municipality of Castro de Aire, utilizing MHUs to reach this population is crucial, given the challenges of accessing healthcare services in Portuguese rural areas. The mobile unit facilitates population-based screening for chronic diseases and enables patient follow-up, complementing existing community-based primary healthcare systems. Dolan et al. [21] highlighted the importance of home ambulatory blood pressure monitoring over clinic blood pressure measurements to predict mortality. In clinical practice, home ambulatory monitoring is a better tool for diagnosing and titrating medications. These MHUs might symbolize how close we need to follow the older adult population in rural areas.

The lifestyles presented by the population under study are varied. Regarding alcohol consumption, 45.5% of the sample have never consumed alcoholic beverages, and alcohol dependence is present in 7.2% of those who drink alcoholic beverages. Although most participants did not consume alcohol and alcohol consumption is a risk factor for AHT [22], the frequency of hypertense patients consuming alcohol is higher herein than found in other studies. The study by Ferreira et al. [20] presents similar results, in which 54.6% of the respondents do not drink alcohol. In a cross-sectional study conducted in Brazil with 789 individuals, alcohol consumption is found in 4.7% of people with arterial hypertension [23]. Tobacco consumption is a modifiable risk for AHT [22], and in our study, it was found in 0.9% of the respondents, only among males. The smoking prevalence found in our study was lower than found in a previous Portuguese study of 108 people with arterial hypertension, in which smokers comprised 8.4% of the respondents [24]. The WHO recommends not smoking or using tobacco to treat and prevent cardiovascular diseases such as hypertension [2,22].

The intensity of physical activity practiced by the respondents was evaluated through the IPAQ. As a result, 60.0% practice high-intensity physical exercise, 22.1% moderate intensity, and 17.9% low intensity; that is, they are sedentary. The results presented by Ferreira et al. [20] regarding hypertense participants in Portugal are quite different, with 47.0% admitting low physical activity, 26.2% moderate physical activity, and 25.9% high physical activity. In a study conducted in Brazil, with a sample of 182 hypertensive individuals, the IPAQ results reveal that 22.0% were sedentary, 77.2% practiced moderate physical activity, and only 1.6% practiced high physical activity [25]. It is important to emphasize that the WHO recommends at least 150 min of moderate-intensity aerobic activity or 75 min of vigorous aerobic activity per week and muscle-building exercises more than twice a week to prevent and treat ATH [22]. Compared to other studies, our sample seems to practice more high-intensity physical exercise than the others.

The nutritional health determination questionnaire revealed that 63.4% of participants had good nutritional health. Regarding the preparation of meals, 78.3% of the sample always use salt, while aromatic herbs are only used by 3.4% of the participants. On the contrary, only 2.6% say they never use salt and 49.4% never use aromatic herbs to prepare meals. The study by Ferreira et al. [20] presented a figure of salt consumption in meal of 96.1% of the respondents. In another study [26], with a sample of 120 Portuguese hypertensive participants, 5.8% did not use any salt in their food during the last week, 43.3% rarely used it, 33.3% used it sometimes, 12.5% used it almost every day, and 5% used it every day. Another study [27], in a sample of 61 Portuguese hypertensive individuals, showed that 50.82% of the participants use salt to season food. It is important to emphasize that evidence suggests certain herbs and spices can reduce blood pressure in hypertensive patients and salt tends to increase blood pressure [28,29]. In this sense, the use of aromatic herbs must be stimulated for partial or total salt replacements for hypertense patients [28].

The results of the Self-Care with Hypertension scale varied according to the subscale. For the general dietary subscale, a minimum value of 0.67 days and a maximum of 7 days (M = 4.74 ± 1.39) was observed. For the specific dietary subscale, the minimum was 0.75 days, and the maximum was 4 days (M = 2.18 ± 0.55). The subscale of physical activity had a minimum value of 0 days and a maximum of 7 days (M = 3.34 ± 2.38). In the subscale of intake of antihypertensive medication, the minimum value was 1 day and the maximum was 7 days (M = 6.67 ± 0.92). One study [11] shows a minimum value of 0 days and a maximum of 7 days (M = 4.83 ± 1.72) for the general dietary subscale; for the specific dietary subscale, a minimum of 0.88 days and a maximum of 7 days (M = 5.45 ± 0.95); and for the physical activity subscale, a minimum value of 0 days and a maximum of 7 days (M = 2.70 ± 2.46). Therefore, it is concluded that the results are similar for general diets, discrepant in the specific diet subscale and approximate in the physical activity subscale. Regarding the control of blood pressure levels, it was observed that 34.5% of the sample had controlled blood pressure, 28.2% of males and 38% of females. There are several studies on the prevalence of AHT control. Similar results are found in the study by Macedo and Ferreira [30], presenting an epidemiological analysis in primary healthcare that reveals a prevalence of AHT control in 35.6% of people (33.1% in males and 37.4% in females). In the PHYSA study, the prevalence of AHT control was 55.7%, with the highest percentages of AHT control observed in females [7].

The PAP study on the prevalence, knowledge, treatment, and control of hypertension in Portugal presents a prevalence of AHT control in 28.6% of people, being higher in females (32.1%) than in males (23.4%) [5]. All analyzed studies corroborate the present one by showing that females best control their blood pressure. Regarding treatment adherence, 56.2% of the sample adhere to the therapeutic measures. Other studies have similar results. In the study of Amaral et al. [31], the percentage of treatment adherence is only 26.4%. Whilst one study [27] showed adherence in 50% of the participants, another study [16] revealed that more than 60% were adherent. In the study by Amaral et al. [31] with 106 hypertensive individuals, 50.9% of the male gender, with a mean age of 58.26 ± 11.60 years, treatment adherence was associated with people who always followed medical recommendations (*p* < 0.001) and dietary recommendations (*p* = 0.32) and who rarely practiced physical exercise (*p* = 0.41). The results of the cross-sectional study by Girotto et al. [3] conducted in Brazil with 385 hypertensive patients aged between 22 and 79 years (M = 58.9 years ± 12.0), 62.6% of which were female, showed that the adherence to the pharmacological treatment was 59%. The lifestyles associated with treatment adherence had to do with regularly not consuming alcoholic beverages, not smoking or being an ex-smoker, and the regular practice of physical activity.

In our study, females were more active, had controlled blood pressure, had lower smoking habits, and had better nutritional health than males, more frequently using aromatic herbs and consuming less alcoholic beverages, sausages, and smoked ham. Our study did not explore the reasons, but it could be due to more health concerns and hypertension awareness among females than males [32,33,34]. In contrast, males adhered more to the treatment and reported a lower frequency of salt consumption. This result is similar to that found in an Italian study about differences in medication consumption of hypertensive patients in which females presented lower therapeutic adherence than males [35].

The study has some limitations that should be taken into account. Firstly, its cross-sectional design and the reliance on a small convenience sampling size of only one MHU acting in a specific region of Portugal may limit the generalization of its findings. Secondly, the lengthy questionnaire may have introduced respondent fatigue, which could have impacted the accuracy of the data collected. Finally, the absence of specific information regarding the medication prescribed for hypertension in our study is noteworthy. While our investigation focused on lifestyle factors and treatment adherence among hypertensive patients attending an MHU, the specific pharmacological interventions utilized were not detailed. Understanding the medication regimens employed is important for comprehensively assessing hypertension management strategies. Future research endeavors should incorporate information regarding the types of medications prescribed, dosages, and adherence levels to pharmacotherapy. This additional insight would provide a more comprehensive understanding of the multifaceted approach required for effective hypertension control. Moreover, investigating the interplay between lifestyle modifications and pharmacological treatments could offer valuable insights into optimizing hypertension management protocols and improving patient outcomes. Despite these limitations, the study provides valuable insights into hypertension management and highlights the importance of developing tailored interventions to meet the needs of older adults in rural areas.

## 5. Conclusions

Our study showed that more than half of elderly Portuguese hypertense patients attending an MHU in a rural area of the center of Portugal adhere to therapeutic measures. However, of the total hypertensive patients, only 34.5% had controlled blood pressure. Positive correlations were obtained with the general dietary, antihypertensive medication intake, and physical activity subscales. In contrast, the specific dietary subscale had a negative correlation. Regarding the other lifestyles analyzed, there were no statistical differences. Intervening in health education for people with AHT is a priority to minimize the onset of cardiovascular diseases and complications of AHT. Hence, it is necessary to improve adherence to treatment in its various dimensions. The difficulty in adhering to healthier lifestyles calls into question treatment adherence. Improving treatment adherence, especially concerning physical activity, dietary care, and reduction or extinction of addictive behaviors (abusive alcohol consumption and smoking), could increase the effectiveness of AHT treatment. Thus, one must intervene to improve adherence to non-drug treatments, with a more effective control of blood pressure values, and by maximizing the effects of the prescribed medication. Adopting healthy lifestyles, aimed at effective health promotion, is an objective that is based on international guidelines and the national health plan, as well as on health programs and institutional projects.

Therefore, personalized health education, as well as the involvement and empowerment of the citizen for the management of arterial hypertension, is required. Encouraging individuals to acquire these skills allows them to gain a greater hold on the determinants of their own health, which is a starting point for behavioral change and instituting new lifestyles.

## Figures and Tables

**Table 1:** Socio-demographic characterization of the sample.

Gender	Male		Female		Total	
Variables	n (85)	% (36.2)	n (150)	% (63.8)	n (235)	% (100)
**Age Group**						
≤64	6	7.0	19	12.6	25	10.6
65–75	31	36.5	73	48.7	104	44.3
≥76	48	56.5	58	38.7	106	45.1
**Marital Status**						
Single	3	3.5	8	5.3	11	4.7
Married/Civil Partnership	67	78.8	80	53.4	147	62.5
Divorced/Separated/Widowed	15	17.7	62	41.3	77	32.8
**Academic Qualifications**						
Does not know how to read/write	13	15.3	48	32.0	61	25.9
Knows how to read/write	11	13.0	19	12.6	30	12.8
1–4 years of school	58	68.2	79	52.7	137	58.3
≥5 years of school	3	3.5	4	2.7	7	3.0
**Employment status**						
Employed	2	2.3	1	0.7	3	1.3
Unemployed	6	7.1	17	11.3	23	9.8
Retired	77	90.6	129	86.0	206	87.6
Other	-	0.0	3	2.0	3	1.3
**Residence Area**						
Town	-	0.0	-	0.0	-	0.0
Village	85	100.0	150	100.0	235	100.0

**Table 2:** Participants’ characteristics regarding hypertension management, physical activity, smoking habits, treatment adherence, and nutritional health.

	Gender	Male		Female		Total	
		n (85)	% (36.2)	n (150)	% (63.8)	n (235)	% (100.0)
Hypertension	Controlled	24	28.2	57	38.0	81	34.5
	Not controlled	61	71.8	93	62.0	154	65.5
Categories of physical activity	Low	17	20.0	25	16.7	42	17.9
	Moderate	19	22.4	33	22.0	52	22.1
	High	49	57.6	92	61.3	141	60.0
Smoking habits	Has never smoked	59	69.4	149	99.3	208	88.5
	Over 2 years ago	21	24.7	1	0.7	22	9.4
	1 to 2 years ago	3	3.5	-	0.0	3	1.3
	Currently	2	2.4	-	0.0	2	0.9
Adherence to treatment (MAT)	Adherent	50	58.8	82	54.7	132	56.2
	Not adherent	35	41.2	68	45.3	103	43.8
Nutritional Health	High nutritional risk	14	16.5	14	9.3	28	11.9
	Moderate nutritional risk	24	28.2	34	22.7	58	24.7
	Good nutritional health	47	55.3	102	68.0	149	63.4

**Table 3:** Characterization of the type of diet and food pattern according to gender.

	Gender	Male		Female		Total		Residuals	
Variables		n (85)	% (36.2)	n (150)	% (63.8)	n (235)	% (100.0)	Male	Fem
**Type of Diet**									
Unhealthy		-	0.0	-	0.0	-	0.0		
Not very healthy		15	17.6	22	14.7	37	15.7	0.6	−0.6
Moderately healthy		58	68.2	107	71.3	165	70.2	−0.5	0.5
Healthy as recommended		10	11.8	19	12.7	29	12.3	−0.2	0.2
Very healthy		2	2.4	2	1.3	4	1.7	0.6	−0.6
**Seasoning**									
**Use of aromatic herbs**									
Never		44	51.8	72	48.0	116	49.4	0.6	−0.6
Rarely		11	12.9	22	14.7	33	14.0	−0.4	0.4
Sometimes		20	23.5	32	21.3	52	22.1	0.4	−0.4
Frequently		8	9.4	18	12.0	26	11.1	−0.6	0.6
Always		2	2.4	6	4.0	8	3.4	−0.7	0.7
**Use of salt**									
Never		2	2.4	4	2.7	6	2.6	−0.1	0.1
Rarely		1	1.2	-	0.0	1	0.4	1.3	−1.3
Sometimes		2	2.4	-	0.0	2	0.9	1.9	−1.9
Frequently		17	20.0	25	16.7	42	17.9	0.6	−0.6
Always		63	74.1	121	80.7	184	78.3	−1.2	1.2
**Food and Beverages**									
**Coffee**									
Never		31	36.5	93	62.0	124	52.8	−3.8	3.8
Rarely		7	8.2	15	10.0	22	9.4	−0.4	0.4
Sometimes		24	28.2	14	9.3	38	16.2	3.8	−3.8
Frequently		5	5.9	8	5.3	13	5.5	0.2	−0.2
Always		18	21.2	20	13.3	38	16.2	1.6	−1.6
**Alcoholic beverages**									
Never		18	21.2	89	59.3	107	45.5	−5.6	5.6
Rarely		5	5.9	10	6.7	15	6.4	−0.4	0.4
Sometimes		12	14.1	12	8.0	38	16.2	3.8	−3.8
Frequently		20	23.5	15	10.0	35	14.9	2.8	−2.8
Always		30	35.3	24	16.0	54	23.0	3.4	−3.4
**Sausages**									
Never		16	18.8	49	32.7	65	27.7	−2.3	2.3
Rarely		43	50.6	71	47.3	114	48.5	0.5	−0.5
Sometimes		21	24.7	26	17.3	47	20.0	1.4	−1.4
Frequently		5	5.9	4	2.7	9	3.8	1.2	−1.2
Always		-	0.0	-	0.0	-	0.0		
**Smoked ham**									
Never		16	18.8	64	42.7	80	34.0	−3.7	3.7
Rarely		46	54.1	61	40.7	107	45.5	2.0	−2.0
Sometimes		19	22.4	21	14.0	40	17.0	1.6	−1.6
Frequently		4	4.7	4	2.7	8	3.4	0.8	−0.8
Always		-	0.0	-	0.0	-	0.0		
**Lupin beans**									
Never		47	55.3	105	70.0	152	64.7	−2.3	2.3
Rarely		22	25.9	23	15.3	45	19.1	2.0	−2.0
Sometimes		14	16.5	19	12.7	33	14.0	0.8	−0.8
Frequently		2	2.4	3	2.0	5	2.1	0.2	−0.2
Always		-	0.0	-	0.0	-	0.0		
**Olives**									
Never		20	23.5	34	22.7	54	23.0	0.2	−0.2
Rarely		33	38.8	67	44.7	100	42.6	−0.9	0.9
Sometimes		27	31.8	43	28.7	70	29.8	3.8	−3.8
Frequently		4	4.7	6	4.0	10	4.3	0.3	−0.3
Always		1	1.2	-	0.0	1	0.4	1.3	−1.3

**Table 4:** Results from Self-Care with Hypertension Scale.

	Min	Max	M	S.D.
General dietary	0.67	7	4.74	1.39
Specific dietary	0.75	4	2.18	0.55
Physical activity	0	7	3.34	2.38
Medication	1	7	6.67	0.92

Min: Minimum; Max: Maximum; M: Mean; S.D.: Standard Deviation.

**Table 5:** Relationship between lifestyle habits and treatment adherence.

Lifestyles	Adherence to Treatment		
	Mean Ranks	Statistical Analysis	*p*
**Alcohol dependence**			
Not dependent	63.53	UMW = 460	0.536
Dependent	56.11		
**Aromatic herb consumption**			
Never/Sporadically	116.87	UMW = 3190	0.518
Frequently/Always	124.68		
**Salt consumption**			
Never/Sporadically	153.00	UMW = 702	0.100
Frequently/Always	116.61		
**Nutritional state**			
Good nutritional health	120.58	KW = 0.640	0.726
Moderate nutritional risk	113.36		
High nutritional risk	113.88		
**Physical activity**			
Low	124.26	KW = 2.645	0.266
Moderate	128.02		
High	112.44		

UMV = U Mann–Whitney test; KW = Kruskal–Wallis test.

## Data Availability

The original contributions presented in the study are included in the article, further inquiries can be directed to the corresponding authors.
